# Efficacy of virtual reality-based training programs and games on the improvement of cognitive disorders in patients: a systematic review and meta-analysis

**DOI:** 10.1186/s12888-024-05563-z

**Published:** 2024-02-12

**Authors:** Khadijeh Moulaei, Hamid Sharifi, Kambiz Bahaadinbeigy, Fatemeh Dinari

**Affiliations:** 1 Department of Health Information Technology, Faculty of Paramedical, Ilam University of Medical Sciences, Ilam, Iran; 2 HIV/STI Surveillance Research Center, and WHO Collaborating Center for HIV Surveillance, Institute for Futures Studies in Health, Kerman University of Medical Sciences, Kerman, Iran; 3 Medical Informatics Research Center, Institute for Futures Studies in Health, Kerman University of Medical Sciences, Kerman, Iran

**Keywords:** Virtual reality, Training programs, Games, Cognitive disorders

## Abstract

**Introduction:**

Cognitive impairments present challenges for patients, impacting memory, attention, and problem-solving abilities. Virtual reality (VR) offers innovative ways to enhance cognitive function and well-being. This study explores the effects of VR-based training programs and games on improving cognitive disorders.

**Methods:**

PubMed, Scopus, and Web of Science were systematically searched until May 20, 2023. Two researchers selected and extracted data based on inclusion and exclusion criteria, resolving disagreements through consultation with two other authors. Inclusion criteria required studies of individuals with any cognitive disorder engaged in at least one VR-based training session, reporting cognitive impairment data via scales like the MMSE. Only English-published RCTs were considered, while exclusion criteria included materials not primarily focused on the intersection of VR and cognitive disorders. The risk of bias in the included studies was assessed using the MMAT tool. Publication bias was assessed using funnel plots and Egger’s test. The collected data were utilized to calculate the standardized mean differences (Hedges’s g) between the treatment and control groups. The heterogeneity variance was estimated using the Q test and I2 statistic. The analysis was conducted using Stata version 17.0.

**Results:**

Ten studies were included in the analysis out of a total of 3,157 retrieved articles. VR had a statistically significant improvement in cognitive impairments among patients (Hedges’s g = 0.42, 95% CI: 0.15, 0.68; *p*_value = 0.05). games (Hedges’s g = 0.61, 95% CI: 0.30, 0.39; *p*_value = 0.20) had a more significant impact on cognitive impairment improvement compared to cognitive training programs (Hedges’s g = 0.29, 95% CI: -0.11, 0.69; *p*_value = 0.24). The type of VR intervention was a significant moderator of the heterogeneity between studies.

**Conclusion:**

VR-based interventions have demonstrated promise in enhancing cognitive function and addressing cognitive impairment, highlighting their potential as valuable tools in improving care for individuals with cognitive disorders. The findings underscore the relevance of incorporating virtual reality into therapeutic approaches for cognitive disorders.

**Supplementary Information:**

The online version contains supplementary material available at 10.1186/s12888-024-05563-z.

## Introduction

Cognitive disorders (CDs), also known as neurocognitive disorders (NCDs), can directly or indirectly interfere with the functioning of the cognitive-neural and perceptual systems, either permanently or transiently [1, 2]. These disorders lead to confusion in individuals’ self-awareness and their perception of the world, resulting in various behavioral abnormalities [3]. Also, these disorders severely affect the personal and social life of the patient and reduce their quality of life [3, 4]. Some of the common cognitive disorders are Alzheimer’s disease, forgetfulness, dementia, developmental disorders, movement skills disorders, and cognitive impairment caused by drug use [5, 6].

Despite the different treatment methods that exist for patients with CD, according to the type of disease, unfortunately, there is no definitive treatment to prevent brain damage caused by CD [7]. However, treatments can be used to help people with CD maintain their mental abilities and skills, mitigate the effects of the disease, and perform their jobs more effectively and without difficulties [8, 9]. Currently, due to the prevalence of brain injuries caused by CD and the challenges associated with drug treatments, interest in using non-drug treatments to prevent or reduce the risk of diseases has increased [10, 11]. One of the non-pharmacological treatments is the use of virtual reality (VR) technology, which is currently employed in the control and treatment of various diseases [12]. VR technology separates the individual from external sensory inputs of the surrounding environment. Then, it immerses them in a simulated world different from their current environment and helps by distracting them within that virtual and simulated environment [13]. VR technology can manifest in VR-based education programs and VR-based games. A VR education program integrates VR technology into learning, creating immersive experiences for participants [14]. Utilizing computer-generated simulations, it enhances understanding through realistic scenarios and encourages active participation in the learning process [14, 15]. VR-based games also involve the use of virtual reality technology to create interactive and immersive gaming experience [16]. In these games, players are fully immersed in a computer-generated environment that responds to their actions in real-time [17]. Education programs leverage VR technology to create immersive learning experiences, utilizing computer-generated simulations to deepen understanding and encourage active participation in educational content [15]. In contrast, VR-based games use VR to provide users with engaging and entertaining experiences, prioritizing narrative, gameplay, and overall enjoyment [18].

Available evidence shows that VR technology could be beneficial in controlling, managing, and treating CD [19–21].VR-based interventions are vital for patients with cognitive impairment due to their immersive and interactive nature, potentially enhancing cognitive functions [22]. This dynamic and personalized therapy allows for targeted interventions based on individual needs [23]. VR has shown promise in improving cognitive skills, memory, and overall cognitive function in various conditions, including Alzheimer’s disease and dementia [24]. Foloppe et al. [25], showed the effects of VR-based training in increasing the functional independence of Alzheimer’s patients during cooking activities. This enables Alzheimer’s patients to learn cooking again through the utilization of VR techniques. In general, it was shown in this study that the improvement of work performance will remain constant over time for patients with Alzheimer’s disease. In a clinical trial study on Alzheimer’s patients, Serino et al. [26], showed that subjects in the intervention group experienced a significant improvement in long-term memory recall after undergoing VR-based training. The findings of this study also showed that training based on VR had a significant effect on the executive performance of cognitively healthy elderly people. Rosa et al. [27], showed that VR-based game interventions allow faster and more effective improvement of several cognitive abilities for patients with cognitive disorders than traditional interventions. Also, Zhu et al. [28], showed in their study that virtual reality as a non-pharmacological treatment can improve cognitive and motor performance in elderly people with mild cognitive impairment or dementia.

To the best of our knowledge, no systematic review or meta-analytic study has been conducted to investigate the effect of educational programs and games based on VR on the improvement of cognitive disorders. Only in some studies, such as Zhang et al.‘s meta-analysis [29], the effect of VR-based treatments on cognition and mental health of stroke patients has been shown. In another meta-analysis study, Yan et al. [30], examined the effects of VR combined with cognitive and physical interventions on cognitive performance in the elderly with mild cognitive impairment. In a review study and meta-analysis, Dan Yua et al. [31] also investigated the effect of virtual reality on executive function in the elderly with mild cognitive impairment. None of these studies focused on all cognitive disorders in general, and only on a specific population such as patients with stroke or the elderly with CD. Furthermore, none of these studies have specifically investigated and analyzed the effect of games and programs based on VR separately. Therefore, the purpose of the present study was to investigate the effect of games and educational programs based on VR on the improvement of cognitive disorders.

## Methods

### Study design

The review adhered to the Preferred Reporting Items for Systematic Reviews and Meta-Analyses (PRISMA) guideline. Ethical approval was deemed unnecessary for its completion (Appendix A).

### Data sources and search strategy

A comprehensive search was conducted in the electronic databases PubMed, Scopus, and Web of Science, covering publications in English from their inception up until May 20, 2023. PubMed, Scopus, and Web of Science are chosen for health-related investigations due to their extensive scope, multidisciplinary character, and reliable indexing of academic publications [32, 33]. PubMed focuses on biomedical and life sciences research, Web of Science is renowned for its meticulous journal curation and citation analysis [33], and Scopus provides comprehensive coverage of scientific literature, spanning both medical and non-clinical sciences. These databases serve as valuable resources for interdisciplinary studies, offering researchers access to a diverse and credible array of scholarly materials. This diverse selection forms a robust foundation for conducting health-related research [32].

The search strategy was collaboratively developed by two researchers, KHN, and FD. After its development, the strategy was then reviewed and approved by KB and HSH. Keywords and search strategies are included:

- ((“virtual reality” OR “virtual reality training " OR " game” OR “gaming” OR “video games” OR “augmented reality”) AND (“cognitive impairment” OR “cognitive disorder” OR “memory disorder” OR “cognitive decline” OR “memory impairment” OR “cognitive dysfunction”))

### Eligibility criteria

Some specific inclusion criteria were established to ensure the relevance and uniformity of the data. Firstly, eligible studies had to encompass individuals with any cognitive disorder, ensuring a focus on the target population. Secondly, participants in each study were required to have engaged in at least one VR-based training session, ensuring familiarity with the technology. Thirdly, the reported results had to contain data on cognitive impairment, assessed through scales like the Mini-Mental State Examination (MMSE), thereby providing a consistent measure of cognitive function. Fourthly, to maintain consistency in language and accessibility, only studies published in the English language were considered. Lastly, only randomized clinical trial (RCT) studies were included. Hernandez et al. [34], pointed out that meta-analyses are commonly classified into two types: traditional and nontraditional. Traditional meta-analyses evaluate the effects of one intervention compared to another (e.g., investigational intervention, usual practice, placebo) by synthesizing aggregated data from prior studies. They combine various study types, including RCTs, observational studies (e.g., cohort studies, case-control studies), diagnostic studies, and prognostic studies. Meta-analyses specifically focused on RCTs are considered the optimal approach for summarizing the positive and adverse effects of interventions [34].

The exclusion criteria encompassed articles that did not primarily focus on the intersection of virtual reality and cognitive disorders. Additionally, materials such as books, book chapters, letters to the editor, and conference abstracts were excluded from the analysis.

### Study selection

Three authors (KHM, KB, and FD) independently reviewed the titles and abstracts of studies obtained from the database searches. They assessed each study against two criteria: (1) whether the study utilized virtual reality technology, and (2) whether the participants were described as individuals diagnosed with any cognitive impairments.

Next, the same three authors conducted a full-text review of the identified studies, evaluating them against the complete inclusion criteria (as specified in the eligibility criteria). In case of any disagreements regarding the inclusion of studies, a fourth reviewer (HSH) was consulted to reach a consensus.

### Data collection and extraction process

To ensure accurate and systematic data extraction, the research team devised a data extraction form to code the demographic, methodological, and outcome variables obtained from each study. The responsibility of extracting the data was carried out independently by KHM and FD. The final dataset, which was to be included in the analysis, was confirmed by both KB and HSH to ensure its accuracy and reliability.

A data extraction form was employed to extract relevant data from the primary studies. This form facilitated the systematic collection of information required for the analysis. The data extraction form encompassed various fields, including the country, population diagnosis, VR equipment, sample size (male/female), mean age, intervention in treatment group, intervention in control group, duration of the session and the follow-up period (Table 1), and mean and standard deviation (SD) of MMSE for experimental and control groups (Fig. 2). In instances where disagreements arose regarding the extracted information, the research team members convened to deliberate and reach a final decision. The extracted information was entered and organized in an Excel spreadsheet.

**Table 1:** Characteristics of the included studies to assess the effect of virtual reality-based training programs and games on the improvement of cognitive disorders in patients

Ref	Country	Population diagnosis	VR Equipment	Sample size (M/F)	Mean age(y)	Intervention in treatment group	Intervention in control group	Duration of the session and the follow-up period
Optale, 2020 [43]	Italy	Older adults with memory deficits	Headphones, joystick, computer	31(21/10)	CG = 81.6 ± 5 EG = 78.5 ± 10.9	VR-based training programs (VR memory training (VRMT) with auditory stimulation and pathfinding experiences)	Face-to-face training sessions using music therapy	45-minute session, 10 sessions in a 4-6-week period, with two sessions per week.
Tarnanas, 2014 [44]	Greece	Older subjects with MCI	A computer	78 (28/50)	CG = 70.5 ± 4.3 EG = 69.7 ± 4.4	Game (virtual reality museum a)	DVD-based educational programs for memory improvement	Twice a week for 90 min across 5 months
Oliveira, 2021 [45]	Portugal	Older adults with Alzheimer	A 17-inch laptop	17(5/12)	CG = 84.14 ± 6.30 EG = 82.60 ± 5.42	VR-based training programs (Utilizing a computerized cognitive stimulation program with non-immersive VR, featuring exercises that simulate instrumental activities of daily living (IADL))	Receiving treatment-as-usual at care units for older adults	10 sessions (two sessions/week, 45 min for each session,) for two months
Kim, 2021 [46]	Italy	Older subjects with cognitive impairment	Xbox Kinect platform	20 (3/17)	CG = 64.70 ± 6.83 EG = 68.32 ± 6.32	VR-based training programs (a VR bicycle riding program through Google Maps)	Performing daily tasks	Once-daily sessions lasting 30 min, two days per week, for a duration of 12 weeks
Kang, 2021 [38]	Korea	Older subjects with cognitive decline or MCI	Head-mounted Oculus Rift CV1 display, along with Oculus Touch controllers held in both hands of patients	41(12/29)	CG = 73.28 ± 6.96 EG = 75.48 ± 4.67	VR-based training programs (Undergoing cognitive training with the assistance of a neuropsychologist in a completely immersive VR environment)	Receiving usual therapy, including pharmacotherapy.	Approximately 20–30 min for each session, twice a week, for 1 month.
Park, 2022 [39]	Korea	Older adults with MCI	Joystick, computer	56 (23/33)	CG = 72.04 ± 2.42 EG = 71.93 ± 3.11	VR-based training programs (a VR-based spatial cognitive task crafted to enhance spatial memory (32 sessions)).	A VR-based spatial cognitive task crafted to enhance spatial memory (24 sessions)	56 sessions, 45 min a session, 3 days a week for 8 weeks
Zheng, 2022 [47]	China	Older people with dementia	A Kinect sensor and a console	38 (9/29)	CG = 84.26 ± 5.48 EG = 81.74 ± 5.79	Game (using the Kinect game Fruit Ninja as an exercise intervention to enhance hand-eye coordination and motor skills)	Receiving usual therapy, including	One h/day, 5 days/week for 8 weeks
Yang, 2022 [40]	Korea	Older adults with MCI	An Oculus VR headset (Oculus quest headset) and two wireless hand controllers	66 (9/57)	CG = 72.7 ± 5.6 EG = 72.6 ± 5.4	Game (Four games encompassing various brain domains, such as attention and working memory)	Educational seminars focusing on health-related subjects, including advice on nutrition and exercise for preventing geriatric diseases.	A total of 24 sessions (each VR training session lasted for 100 min) for eight weeks.
Thapa, 2020 [41]	Korea	Older adults with MCI	An Oculus VR headset (Oculus quest headset) and two wireless hand controllers	68 (16/50)	CG = 72.7 ± 5.6 EG = 72.6 ± 5.4	Game (Four varieties of VR game-based content designed to enhance attention, memory, and processing speed.)	An educational program focusing on overall healthcare.	A total of 24 sessions (100 min each session) for eight weeks
Lim, 2023 [42]	Korea	Older adults with MCI	A tablet	24 (7/17)	CG = 73.33 ± 17.52 EG = 75.42 ± 5.74	Game (A Brain Talk™ home-based Serious game comprising 10 sections: memory, thinking, attention, language, mathematics, visual fields, planning, agility, and coordination, audiovisual training)	Performing daily tasks	12 training sessions, each lasting 30 min, conducted three times per week over a period of three weeks.

### Risk of bias assessment

KHM and FD conducted a critical assessment of the data independently, utilizing the Mixed Methods Appraisal Tool (MMAT, version 2018: Hong et al., 2018). The MMAT serves as a crucial assessment instrument tailored for systematic mixed studies reviews, which encompass a range of study types, including qualitative, quantitative, and mixed methods studies. The MMAT is designed to evaluate the methodological quality across five categories of studies, including qualitative research, randomized controlled trials (RCTs), non-randomized studies, quantitative descriptive studies, and mixed methods studies [35]. In cases where there were disagreements between the two authors, KB and HSH intervened to mediate discussions and reach a consensus. The studies were evaluated based on the MMAT criteria corresponding to their respective categories. The most recent version of MMAT presents a descriptive quality appraisal approach rather than using numerical scores. The response options for all study categories include “yes,” “no,” and “can’t tell.” If a study received a “can’t tell” response, it indicates that there was insufficient information reported to provide a definitive “yes” or “no” answer. In such cases, further investigation through companion studies or direct communication with the study authors may be necessary [36].

Articles that fulfilled all five quality criteria were classified as high-quality, earning a full rating of five stars or 100% quality. Additionally, articles meeting four criteria were considered to have 80% quality, those with three stars had 60% quality, two stars indicated 40% quality, and one star denoted 20% quality. If a study did not satisfy any of the quality criteria, it received no stars [37].

### Data synthesis and analysis

The meta-analysis was conducted when several studies reported the effects of using VR on the improvement of cognitive disorders. The standardized mean differences represented by Hedges’s g to compare the treatment and control groups were calculated from the collected data. Publication bias was evaluated using a funnel plot and Egger’s test. The statistical heterogeneity was estimated through the use of the Q test and incoherence index (I2). Moreover, Meta-regression was employed to assess the source of the heterogeneity among included the studies. The effect size calculation involved the utilization of a random effects model for 95% CI. The statistical analysis was performed using Stata version 17.0 software.

## Results

### Study selection

A comprehensive search initially yielded a total of 3157 articles. Following the removal of duplicates, a set of 2556 studies remained. Each of these studies underwent a rigorous and meticulous review, during which inclusion and exclusion criteria were carefully applied. The assessment process involved a thorough examination of the methodologies, results, and relevance to the research focus.

**Fig. 1:**
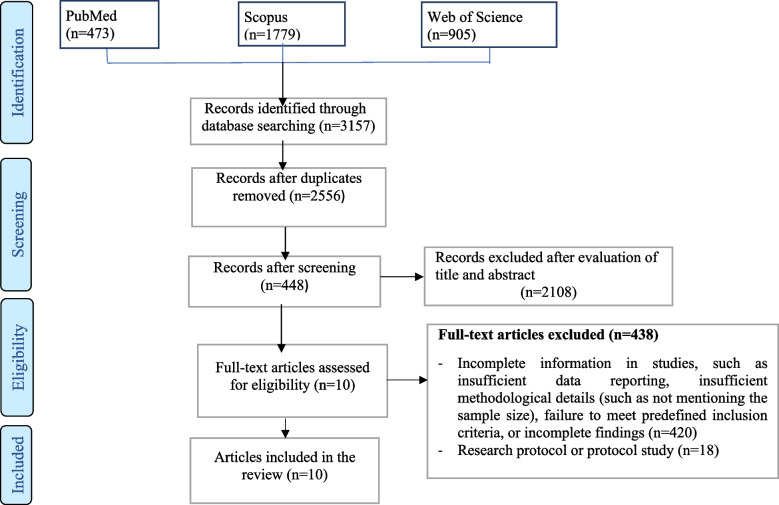
PRISMA flowchart of screened and included studies to assess the effect of virtual reality-based training programs and games on the improvement of cognitive disorders in patients

After this meticulous screening, a total of 10 articles emerged as meeting the predefined criteria for inclusion in the study. The details of this selection process are visually represented in Fig. 1.

### Study characteristics

Table 1 provides a comprehensive overview of the selected studies. As this table shows, most of the studies were conducted in Korea (*n* = 5, 50%) [38–42], and all the included studies were related to elderly people with cognitive disorders. The largest sample size was associated with the study of Thapa et al. [41].

### Quality assessment

Appendix B displays the results of the quality evaluation conducted on the studies using the MMAT tool

### The effect size of included studies

**Fig. 2:**
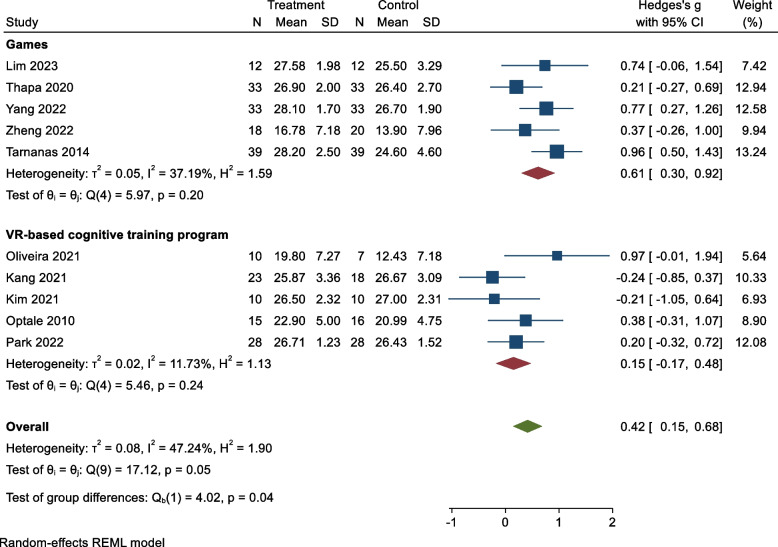
Forest plot of the studies

We found that VR-based interventions including games and training programs have a positive effect on cognitive impairment (Hedges’s g = 0.42, 95% CI: 0.15, 0.68; *p*_value = 0.05). The highest and lowest effect sizes were related to the study of Tarnanas [44] and Oliveira [45], respectively (Fig. 2).

Based on Cohen’s d standardized effect size, this effect size is medium [48]. Also, games (Hedges’s g = 0.61, 95% CI: 0.30, 0.92, *p*_value:0.20) have been demonstrated to improve cognitive disorders more effectively than cognitive training programs (Hedges’s g = 0.15, 95% CI: -0.17, 0.48, *p*_value:0.24) (Fig. 2).

### Publication bias

**Fig. 3:**
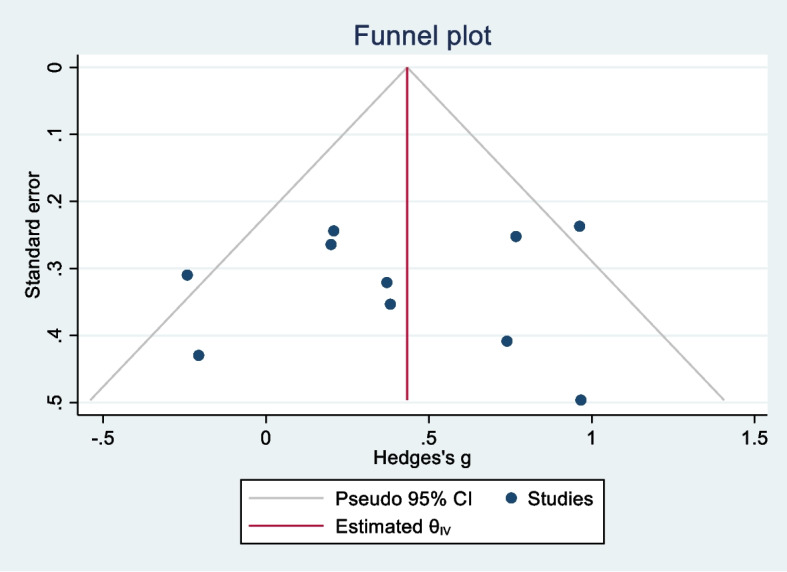
Funnel plot

The funnel plot (Fig. 3) illustrates the absence of publication bias in the studies. Moreover, the output of Egger’s test was not significant. This shows there is no publication bias (*p*_value = 0.86).

### Heterogeneity among included studies

Based on the results of the Q test and a significance level of *P* < 0.001, it can be concluded that the assumption of homogeneity of the studies is rejected with an error rate of less than 1%. This confirms the significance of the Q index, indicating heterogeneity in the effect size across the studies. The value of I^2^ value is also 47.24%, indicating a moderate level of heterogeneity [49].

### Meta-regression analysis

The effect sizes obtained from the random effects model were not found to be statistically significant for gender, age, sample size, duration of intervention, and type of cognitive disorders (*P* > 0.05) (Table 2). This means that these variables did not have a great impact on the results of studies related to the improvement of cognitive disorders through virtual reality. However, the effect size obtained from the random effects model for the type of VR intervention was statistically significant (*P* = 0.047). Therefore, this variable had an impact on the heterogeneity of the included studies (Table 2).

**Table 2:** Effect sizes for separate meta-analyses on moderator variables

Variables (values)	Effect size	I2 (%)	Variance	z-value	*P*-value
**Age (raw values of mean age)**	0.00	52.50	0.1098	0.24	0.683
**Gender (male, female, male and female)**	4.35	47.24	0.085	-1.11	0.350
**Sample size (raw values of sample size)**	10.25	44.43	0.07588	0.93	0.353
**Type of VR intervention (game and cognitive training programs)**	53.19	29.21	0.0395	-1.91	0.047
**Duration of intervention (< 20 h, > 20 h)**	0.00	52.33	0.1075	0.19	0.851
**Type of cognitive disorders (AD and MCI)**	0.00	53.57	0.1052	0.91	0.361

## Discussion

In this study, the effect of VR-based training programs and games on the improvement of cognitive disorders in patients was investigated. Our research revealed that the implementation of virtual reality had a notable and statistically significant impact on enhancing cognitive impairments in patients. Furthermore, the results demonstrated that games were more effective in improving cognitive impairment compared to cognitive training programs.

As stated in our study’s findings, the utilization of virtual reality demonstrated a noteworthy and significant influence in improving cognitive disorders among patients. Different studies [50–54] have also demonstrated that VR-based games and educational programs can enhance the performance of individuals with cognitive disorders. Thapa et al. [55], conducted a study indicating that VR-based educational programs improved cognitive and physical performance in patients with mild cognitive impairments. Similarly, Yang et al.‘s study [56] revealed the potential benefits of virtual reality training for enhancing cognitive performance in brain tumor patients. These studies collectively highlight the potential of VR technology to encourage better treatment adherence among both healthcare providers and patients [57]. By immersing patients in virtual training scenarios and equipping them with innovative tools, VR facilitates a more immersive and engaging learning experience. This can contribute to a deeper understanding of treatment protocols and potentially improve patients’ adherence to treatment processes [58]. Manera et al. [59], concluded in their research that utilizing VR-based training represents an intriguing approach for enhancing adherence to cognitive training among elderly individuals with cognitive impairment. In their study, Then et al. [60], also illustrated that virtual rehabilitation conducted remotely enhances patient motivation, thereby improving therapy adherence. As we navigate the evolving landscape of healthcare, the integration of VR technology holds considerable promise for fostering enhanced treatment adherence and improving outcomes for both healthcare providers and patients. Moreover, Manera et al. [59], further demonstrated that the utilization of VR-based technologies provides patients with mild cognitive impairment (MCI) a sense of safety and satisfaction, reduces fatigue, anxiety, and stress in comparison to alternative educational methods (such as pen and paper-based approaches). Additional research [61, 62] has also highlighted the positive impact of engaging patients through VR-based stimuli or external factors, leading to enhancements in functional and mental abilities, as well as overall quality of life.

The question now arises: why is it that virtual reality technologies can improve cognitive disorders? Some reasons have mentioned how VR technologies could improve CD. Rose et al. [63], highlighted that VR offers an artificial, interactive environment closely mimicking and enhancing reality. VR technologies also offer a heightened degree of resemblance between the educational or gaming environment and the real world. These factors promote stronger patient adherence to their treatment. Additionally, this technology holds greater potential for transferring to daily activities among patients with cognitive impairment. By regulating emotions and providing immediate, precise performance feedback, VR proves to be a favorable method in comparison to alternative approaches. As highlighted by Miedany et al. [64], the engagement of patients in immersive and interactive virtual environments through VR effectively stimulates cognitive processes, fostering improvements that could have significant implications for medical intervention and patient care. In another study [65], it has been noted that virtual reality enables individuals with cognitive impairments, the elderly, and those with Alzheimer’s disease to more readily and securely experience sensory stimuli within a virtual simulation environment, in an easier and simpler manner. This very same virtual environment enhances the comprehension and acquisition of functional learning and function transfer. In connection with this assertion, it should be noted that several studies [15–17] have demonstrated how the virtual environment stimulates and activates brain metabolism, increases cerebral blood flow, facilitates the movement and release of neurotransmitters, and supports various cortical functions. It holds the potential to reactivate and promote brain healing.

The findings of our study revealed that VR-based games have been more effective in improving cognitive disorders compared to VR-based educational programs. Liu et al.‘s study [66] demonstrated significant improvements in the Montreal Cognitive Assessment (MoCA), Trail-Making Test-A (TMT-A), Digit Symbol Substitution Test (DSST), Digital Span Test (DST), Verbal Fluency Test (VFT), and Modified Barthel Index (MBI) following the use of VR-based puzzle games by stroke patients. Yang-Kun et al. [67], demonstrated that VR-based games enhance children’s cognitive performance, including attention, critical thinking, abstract reasoning, and information processing. Yanguas et al.‘s study [68] similarly exhibited cognitive function improvement through the utilization of VR-based games. VR-based games offer users a captivating experience by incorporating features such as interactive screens, touch controllers, motion sensors, voice notifications, physical vibrations, omnidirectional vision, and engaging gameplay elements like collecting points and defending against enemies. These aspects contribute to the attraction, sense of competition, and excitement that users derive from these games [69–71]. It is for this reason that these games have a more significant impact than training based on virtual reality. For example, the feeling of being in places such as bicycle or ski tracks, football fields, or engaging in games such as adventure games and duels, which react extremely quickly, brings a special appeal to users who have Cognitive disorders [72]. Moreover, in some adventure-style games, users manage the game with their own body movements and discover the mystery inside, rather than typing commands or clicking in the game [73]. This itself is very attractive to the user and captivates their attention.

Finally, what is crucial for us to understand is that the design of virtual reality-based training programs and games can significantly influence users’ engagement. Moulaei et al.‘s [18] investigation delves into the scoping review, shedding light on identifying the parameters necessary to design a successful rehabilitation game and the outcomes of using these games. Their findings emphasize the importance of considering specific design elements, such as the immersive nature of virtual reality environments, in achieving positive outcomes. This studies collectively underscore the importance of thoughtful design in virtual reality-based programs and games for optimizing their impact in patients. The synthesis of evidence from various sources informs our understanding of how specific design features contribute to the overall efficacy of virtual reality interventions in the context of cognitive disorders. Additionally, Samarasinghe, et al.‘s research [74], focused on the design aspects of virtual reality games for people with Alzheimer, highlights the significance of tailoring interventions to address the unique cognitive needs of patients. These diverse perspectives collectively reinforce the pivotal role of thoughtful design in maximizing the effectiveness of virtual reality interventions in the realm of cognitive health. Moreover, Understanding the specific cognitive challenges faced by this population informs the tailoring of virtual reality interventions to meet their unique needs, thereby optimizing the potential benefits.

Additionally, unlike conventional cognitive exercises that might feel monotonous or repetitive, games often offer a dynamic and captivating environment that naturally holds the player’s attention [75]. This heightened engagement is significant as sustained focus and interest can foster a more immersive cognitive experience, potentially leading to increased neural plasticity and the strengthening of cognitive pathways. The interactive nature of games, requiring players to make quick decisions, solve puzzles, and adapt to changing scenarios, inherently stimulates a broader spectrum of cognitive functions, effectively providing a comprehensive mental workout [76]. In the other hand, the emotional and psychological involvement elicited by games can contribute significantly to their effectiveness [77, 78]. The enjoyment, achievement, and sense of progress experienced during gameplay can trigger the release of neurotransmitters such as dopamine, which are linked to motivation and reward systems in the brain [79]. This neurochemical response can create a positive feedback loop, encouraging individuals to invest more time and effort into gaming activities. As a result, the increased time spent engaging with games may lead to prolonged cognitive engagement and exposure to diverse cognitive challenges, ultimately facilitating more robust cognitive improvements. These findings shed light on the potential inherent in leveraging the unique qualities of gaming as a promising approach to address cognitive impairments more effectively, marking a paradigm shift in therapeutic interventions.

### Study implication

#### Theoretical implications

The theoretical implications of this study contribute to the growing body of literature exploring innovative interventions for cognitive disorders. By demonstrating the statistically significant improvement in cognitive impairments among patients through VR-based interventions, our findings support the notion that technology, particularly virtual reality, can play a crucial role in addressing cognitive challenges. The identification of games as having a more substantial impact on cognitive impairment improvement compared to cognitive training programs adds nuance to individuals understanding of effective VR interventions. This insight may prompt further research into the specific attributes of VR games that contribute to cognitive benefits.

Furthermore, the identification of the type of VR intervention as a significant moderator of heterogeneity highlights the need for a nuanced approach in assessing and implementing VR-based interventions. Understanding the role of different VR interventions can inform the development of tailored interventions for specific cognitive disorders, thus contributing to the theoretical foundation of personalized cognitive rehabilitation strategies.

#### Practical implications

The practical implications of this study are particularly relevant for clinicians, caregivers, and policymakers involved in the care of individuals with cognitive disorders. The demonstrated promise of VR-based interventions in enhancing cognitive function suggests that incorporating such technologies into therapeutic approaches can be beneficial for patient care. Clinicians may consider integrating VR interventions, especially games, into their treatment plans to complement traditional cognitive training programs.

Caregivers can explore VR-based interventions as a potential at-home therapy option, offering individuals with cognitive disorders an engaging and accessible means of cognitive improvement. Policymakers may find value in supporting the integration of VR technologies into healthcare settings, recognizing their potential to enhance cognitive care and improve overall well-being in individuals with cognitive disorders.

### Limitations

This study has two limitations. Firstly, it only reviewed articles written in English, and it is recommended that future studies encompass articles published in non-English languages for a more inclusive analysis. Secondly, the search for related studies was conducted across three databases. However, to attain more comprehensive results, it is advisable to conduct further research across additional databases.

## Conclusion

In conclusion, our systematic review and meta-analysis revealed a substantial and statistically significant improvement in cognitive function through the utilization of virtual reality interventions. Notably, games emerged as a particularly effective approach, outperforming traditional cognitive training programs in enhancing cognitive abilities among patients. These results underscore the potential of virtual reality as a valuable tool in cognitive rehabilitation strategies and highlight the advantages of incorporating game-based interventions for optimal cognitive outcomes. Further researches and explorations in this domain may provide even more comprehensive insights into the therapeutic applications of virtual reality for cognitive enhancement in clinical settings.

### Supplementary Information

**Additional file 1.**

**Additional file 2.**

## Data Availability

All data generated or analyzed during this study are included in this published article.

## Abbreviations

VR: Virtual reality
MMAT: Mixed methods appraisal tool
CD: Cognitive disorders
KNCD: Known as neurocognitive disorders
MMSE: Mini-mental state examination
RCT: Randomized clinical trial
MCI: Mild cognitive impairment
MoCA: Montreal cognitive assessment
TMT-A: Trail-making test-A
DSST: Digit symbol substitution test, DST:digital span test, VFT:verbal fluency test
MBI: Modified barthel index
