# Supplementary material for: Efficacy of virtual reality-based training programs and games on the improvement of cognitive disorders in patients: a systematic review and meta-analysis
Source: BMC Psychiatry. 2024 Feb 12;24:116. doi: 10.1186/s12888-024-05563-z (PMC10860230; doi:10.1186/s12888-024-05563-z)
Supplement: Supplementary file 2 — Additional file 2. [file 12888_2024_5563_MOESM2_ESM.docx]

| **Quantitative randomized controlled trials** | | | | | | **Score** | **Quality of the article (%)** |
| --- | --- | --- | --- | --- | --- | --- | --- |
| **Ref** | **Is randomization appropriately performed?** | **Are the groups comparable at baseline?** | **Are there complete outcome data?** | **Are outcome assessors blinded to the intervention provided?** | **Did the participants adhere to the assigned intervention?** |  |  |
| Optale, 2020 | Y | Y | Y | Y | Y | ***** | 100 |
| Tarnanas, 2014 | Y | Y | Y | Y | Y | ***** | 100 |
| Oliveira, 2021 | Y | Y | Y | Y | Y | ***** | 100 |
| Kim, 2021 | Y | Y | Y | Y | Y | ***** | 100 |
| Kang, 2021 | Y | Y | Y | Y | Y | ***** | 100 |
| Park, 2022 | Y | Y | Y | Y | Y | ***** | 100 |
| Zheng, 2022 | Y | Y | Y | Y | Y | ***** | 100 |
| Yang, 2022 | Y | Y | Y | Y | Y | ***** | 100 |
| Thapa, 2020 | Y | Y | Y | Y | Y | ***** | 100 |
| Lim, 2023 | Y | Y | Y | Y | Y | ***** | 100 |
| Hong, 2023 | Y | Y | Y | N | Y | **** | 80 |

**Appendix B**: Quality Assessment of Included Studies Using the MMAT Criteria
